# No-vaxxers are different in public good games

**DOI:** 10.1038/s41598-022-22390-y

**Published:** 2022-10-28

**Authors:** Marcello Basili, Alessio Muscillo, Paolo Pin

**Affiliations:** 1 Department of Economics and Statistics, Università di Siena, 53100 Siena, Italy; 2 BIDSA, Università Bocconi, 20136 Milan, Italy

**Keywords:** Human behaviour, Viral infection

## Abstract

In September 2021 we conducted a survey to 1482 people in Italy, when the vaccination campaign against Covid19 was going on. In the first part of the survey we run three simple tests on players’ behavior in standard tasks with monetary incentives to measure their risk attitudes, willingness to contribute to a public good in an experimental game, and their beliefs about others’ behavior. In the second part, we asked respondents if they were vaccinated and, if not, for what reason. We classified as *no-vaxxers* those (around $12\%$ of the sample) who did not yet start the vaccination process and declared that they intended not to do it in the future. We find that no-vaxxers contribute less to the public good in the experimental game because they trust others less to do so. from the three tests we extrapolated a classification based on the benchmark of rationality and other-regarding preferences for each respondent, and we found that in this respect no-vaxxers do not differ from the rest of the population.

## Introduction

In 2020, Italy was hit hard by Covid19and the shock has been large in the public opinion. When the vaccination campaign started in February 2021 the general attitude has been enthusiastic. Nevertheless, by the end of the summer, a substantial part of the population was not adhering to the vaccination campaign, which was still not mandatory , as it became only after February 2022. In the Supporting Information we provide a description of the spread of the disease during that precise period in Italy and across the world.

We conducted a survey in September 2021 on a representative sample of the Italian population. The survey starts with standard behavioral economics games. and, in the second part of the survey (which was unrelated to the first part) included Covid19-related questions. We classify as *no-vaxxers* those who report willingness not to be vaccinated (12% of the sample, while 15% were not vaccinated). The contribution of this paper is to check if there are differences existed between no-vaxxers and the rest of the population in the measures related to behavioral economics. We find indeed that no-vaxxers are statistically significant different in their contribution to the public good in the experimental game. Moreover, we found that they are more prone to believe that the rest of the population is also less willing to contribute than they actually are. However, we found no evidence indicating that no-vaxxers had different levels of risk aversion. This suggests what is the mechanism preventing no-vaxxers from contributing. The current study aims to identify this mechanism by performing statistical analysis and developing a theoretical model.

We are not the first study who try to identify specific features of people who declare to be against vaccines. However, we believe to be the first ones focusing on behavioral economics measures among this group. Such measures are important when we consider adherence to vaccination campaigns, because this decisions involve consideration about risk aversion, trust in others, strategic interaction with others, and willingness to contribute in public goods^1–3^.

## Related literature

While debate exists regarding the optimal methods for promoting COVID vaccination (coercion, incentives or persuasion)^4–6^, recent studies have reached a consensus and highlighted the importance of behavioral science-related insights to support pandemic response and policies^7^. Additionally, previous research has highlighted the role of behavioral economics experiments^8,9^. In line with the previous literature, we found that subjects who behave prosocially in incentivized economic games have greater prevention intentions. In particular, with online surveys and field experiments Jordan and colleagues have studied the importance of framing and show that messages that emphasize both personal and public benefits of prevention have a greater impact on fostering prevention behavior intentions^10^. In another survey study, Campos-Mercade and colleagues found that people are generally averse to exposing others to risks for their own benefit and that this social preference measure is related to health behaviors during the pandemic^11^. With a slightly different point of view, an incentivized experiment by Agranov and colleagues^12^ focuses instead on how others affect individual decisions and, specifically, the intention to be vaccinated. While the authors are able to link the likelihood of being vaccinated to beliefs about others’ behavior and one’s own socioeconomic and personal characteristics, our approach is different because we use behavioral experiments and measures that are unrelated to the pandemic.

## Brief description of survey and games

The survey has been run on a representative sample of the Italian population. We provide more information and descriptive statistics in the Methods section. The main body of the survey consisted of 3 games and a short list of Covid19-related questions. The games are incentivized with monetary rewards. So, on top of a show-up fee, respondents are paid on the basis of the outcome they obtain in the games played.

**Figure 1:**
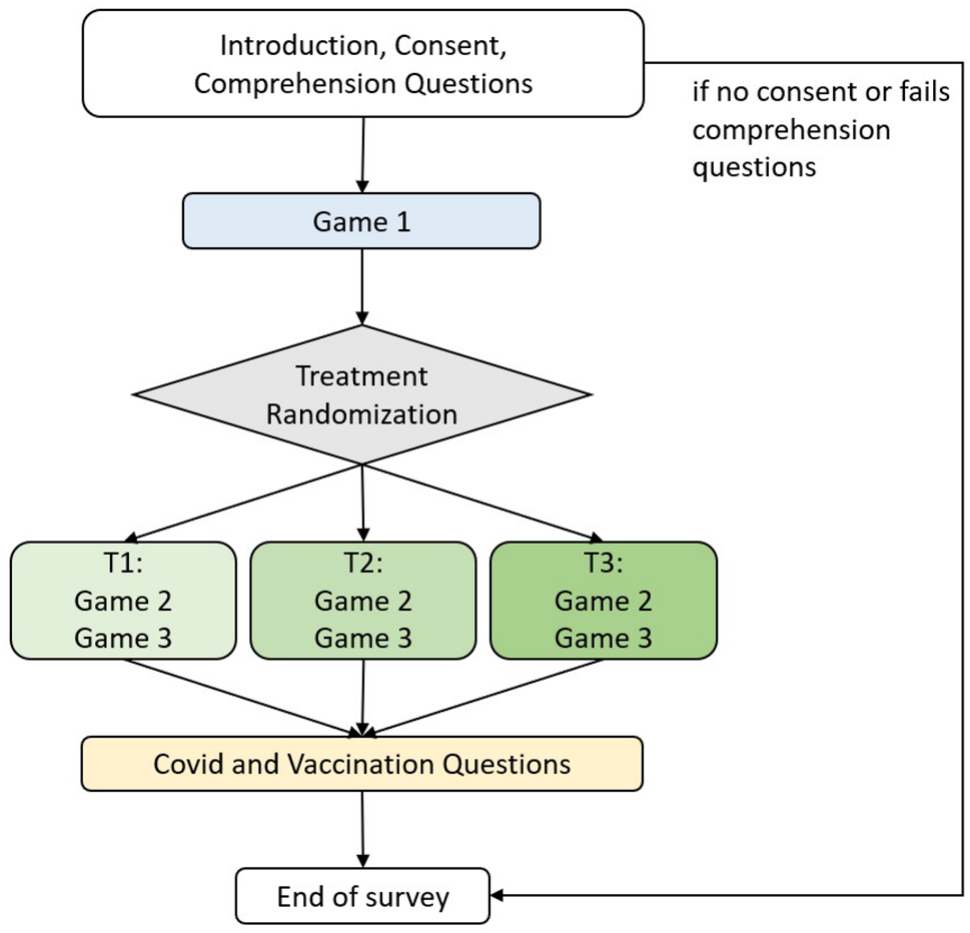
Survey flow. After introduction and comprehension questions, the survey proceeds with Game 1, which is a risk elicitation game. Then individuals are randomly assigned to their treatment T1, T2 or T3 all consisting of a variation of a public good game (Game 2) and a belief elicitation game about the others’ behavior (Game 3). The survey ends with Covid- and vaccination-related questions.

The survey flow is described in Fig. 1. In the beginning, respondents face a comprehension test to check their level of attention: only those who answered correctly are allowed to continue the survey, so we end up with 1,482 individuals. In the Supporting Information, we show that this does not introduce a *selection bias*.

The games are as follows. Game 1 is a *risk elicitation game*^13^, where respondents are given 200 tokens (each token is worth one Euro cent) and are asked the amount $q \in [0,200]$ that they want to invest in a nonrepayable project yielding 2.5*q* with probability $50\%$ and 0 otherwise. In either case, the respondent keeps what is not invested, i.e. $200-q$. Game 2 is a (one-shot binary threshold) *public good game*^14^, where respondents are told that they are grouped with $N-1$ other respondents and that they have the possibility of contributing (or not) to a project to produce a good that will benefit everyone, including those who did not contribute. The project will be executed only if the number of those who choose to contribute meets or exceeds a known threshold $T<N$. Otherwise, the project is not executed and the public good is not produced. In this game, individuals have the incentive to free ride on the others’ contribution to the project. The respondent’s possible actions and payoffs are summarized in Table 1. Game 3 is a *belief elicitation game*, where every respondent is asked how many in her group she thinks had contributed in Game 2. The payoff depends on how close the guess is to the actual number of contributors and is described in Table 1.

After Game 1, individuals are randomly assigned to one of the following three variants of Game 2. Treatment 1 (T1), called “small and risky”, is such that in Game 2 the groups are of $N=5$ people and the threshold is $T=3$. Moreover, in this case, if the number of contributors does not meet nor exceed the threshold, then those who decide to contribute will lose their investment. Treatment 2 (T2), called “large and risky”, is like T1, with the variation that the groups are now of $N=50$ people and the threshold is $T=25$ individuals. Treatment 3 (T3), called “large and safe”, is such that groups are of $N=50$ people and the threshold is $T=25$ individuals, as in T2. However, in this treatment if the threshold is not met or exceeded, those who decided to contribute will get an individual compensation (instead of totally losing their investment, as in the ‘risky’ cases of T1 and T2), so that contributing in Game 2 is a ‘safer’ choice with respect to T1 and T2.

We split our sample into three treatments for Game 2 for two reasons. The first is to test robustness against different variants of this public good game. The second set of reasons is to relate the behavior in these games with vaccination behavior and intentions. We believe that the different treatments can resemble different perceptions about costs, uncertainty and reward that are present when deciding upon vaccination. Indeed the difference in group size ($N=5$ in T1 and $N=50$ in T2 and T3) can highlight the perception of a smaller/larger community that has to contribute and is able to exceed the threshold *T* which can be thought of as a *herd immunity threshold* to be reached. The presence of a safe component (T3, as opposed to a completely risky game like T1 and T2) is meant to represent the fact that the vaccine is used to prevent individual infection or only prevent others’ contagion once one is infected (in which case, vaccination is a pure public good).

**Table 1:** Payoff’s structure of Game 2 (a) and Game 3 (b).

	${}<T$	${}\ge T$
**(a)**		
Contribute	0*, 200**	300
Not contribute	100	400

Guess in G3		
**(b)**		
Exact	300	
Error of $\pm \, 1^\star$ ($2^{\star \star }$)	150	
Error of $\pm \,2^\star$ ($10^{\star \star }$)	100	
Larger error	50	

(a) Game 2: payoff to a subject in the case that she contributes or not, depending on the other players exceeding or not the threshold *T*. The numbers are in tokens. One asterisk (*) for payoff in treatment T1 and T2, while two asterisks (**) for T3.

(b) Game 3: payoff to a subject. It depends on how close the respondent’s guess is to the actual number of others in her group who contributed. The numbers are in tokens. One star ($\star$) for payoff in treatment T1 and two stars ($\star \star$) for T2 and T3.

As shown in the diagram in Fig. 1, after the games are played, respondents are asked a series of Covid19- and vaccination-related questions. Crucially, the games are not framed in a way that can recall or refer to the pandemic situation, to Covid19, to vaccines or even to the notion of public goods. So, this last part was unexpected for the subjects. In this last part of the survey, we ask whether they are vaccinated or not and, if not, whether they are temporarily exempted because they already caught Covid19, whether they do not want to get vaccinated or instead they were not able to get vaccinated yet but they want to get the shot in the future.

We then categorize as *no-vaxxers* as those respondents who are not vaccinated, report not to have had Covid19, and say that they do not want to get vaccinated. They amount to $12.1\%$ of the sample. Notice that those who are not vaccinated are around $15\%$ of the sample had not been vaccinated. These figures are in line with the estimation provided by the Italian government of 7.5 million people not vaccinated in Italy at the time of the survey (September 2021)^15^. Notice also that at the time of the survey vaccination was not mandatory.

## Results

**Figure 2:**
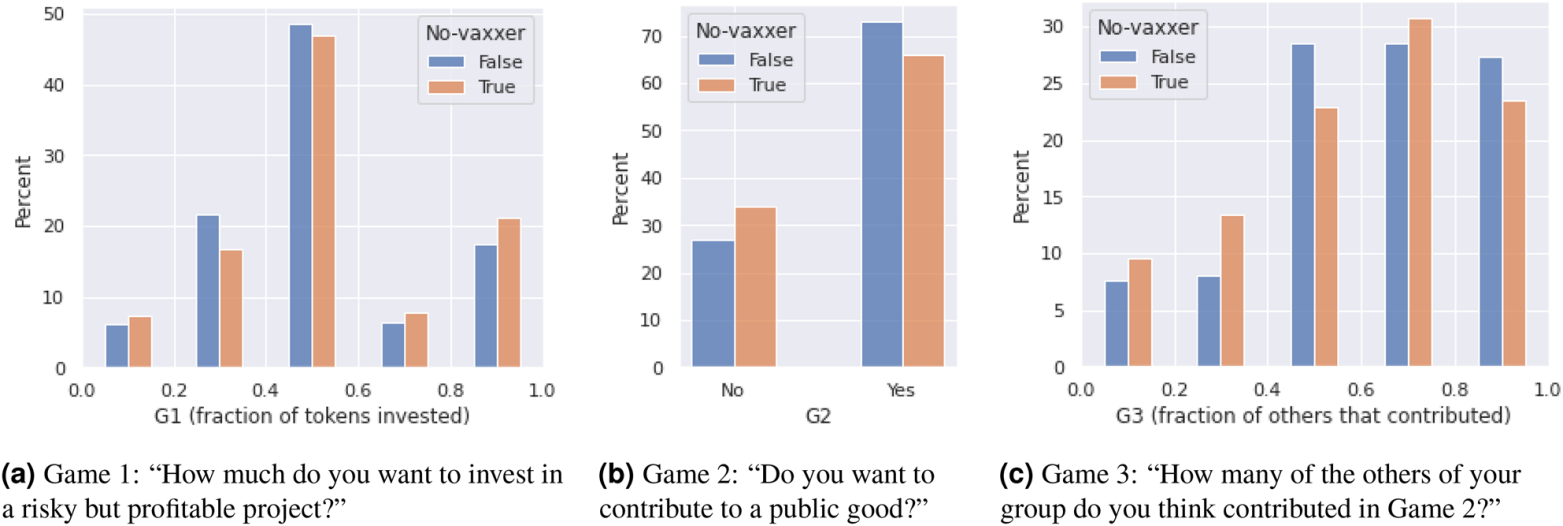
In panel (**a**), on the x-axis the fraction of tokens that respondents choose to invest in Game 1, with the rest of tokens not invested remaining to them. In panel (**b**), the percentage of respondents choosing to contribute to a public good in Game 2 (73% of vaxxers, 65.9% of no-vaxxers). In panel (**c**), the results of Game 3, i.e., on the x-axis respondents’ belief about the fraction of others’ contributing in Game 2.

Results of Game 1 are shown in Fig. 2a. As confirmed in the previous literature^16,17^, the majority of respondents are risk averse because they invested no more than half of their tokens, i.e., 100 tokens or less of the 200 available to them. No-vax individuals tend to invest more than the others, thus suggesting that they are less risk averse. However, this difference is not statistically significant. Indeed, the *p* value of the $\chi ^2$ test on the whole distribution is 0.746—moreover, when investments are classified in above and below 100 tokens, the *p* value of the Barnard test is 0.105. n what follows, in $2\times 2$ cases we will use the Barnard test as opposed to the Fisher test, in line with the recent literature^18,19^.

Results of Game 2, where respondents choose whether to contribute or not in a public good game, are shown in Fig. 2b. This figure shows that no-vax individuals tend to contribute less than the others to the public good game, by approximately 7%, and that this difference is statistically significant at 5% (*p* value: 0.048).

In Game 3, respondents are asked to guess how many other respondents in their group they think contributed to the public good game (Game 2). The results shown in Fig. 2c seem to suggest that no-vaxxers believe that the others’ contribution will be lower than what is expected by the other individuals and, additionally, it also shows that it is lower than the actual contribution of others. However, this result is not statistically significant (but in T2, as fully reported in the Supporting Information).

From these games, assuming full rationality in the sense of *homo economicus*^20^, we construct a classification of the level of other-regarding preferences that would imply contribution. From Game 1, we assess the level of risk aversion of an individual. From Game 3, we assess a range for the i.i.d. probability that they attribute to another subject contributing. From this probability, we can compute the individual’s estimate that the threshold will be reached and, hence, that the public good is realized, independently of her contribution. Thus, a rational selfish agent should contribute only when she (thinks that she) is pivotal, namely, when the number of others contributing is exactly $T-1$ and, so, when with her contribution the public good is made. We classify all our subjects in three types: (*i*) those who are consistent with the behavior of a rational selfish agent; (*ii*) those who are consistent with a rational agent with altruistic behavior (i.e., positive other-regarding preferences); and (*iii*) those who are consistent with a rational agent with negative other-regarding preferences. For robustness, in the Supporting Information we build a parameter to describe a player’s other-regarding preferences and check the consistency with this classification.

On the basis of this classification, we find that no-vaxxers are not statistically different from the rest of the population (full details in the Supporting Information). This classification suggests that the majority of respondents are willing to forego their own outcome to benefit others’ outcomes (around 65%, of type (*ii*)), while around 30% of respondents behave as selfish agents (30%, of type (*i*)). The motivations for such behaviors do not differ between vaxxers and no-vaxxers, even if it seems that slightly more vaxxers are of type (*i*).

### Statistical analysis

For a statistical analysis of our data we proceeded in two phases. First, we performed a regression to assess the relationship between being a no-vaxxer and the belief about others’ behavior. Then, we performed a second regression where we used how an individual has played in Game 1 and Game 3 as regressors and assessed the relationship with her contribution in Game 2. The descriptive statistics of the variables considered are shown in Table 2.

More specifically, we first performed the following ordinary least square (OLS) regression

1 $$\begin{aligned} \text {G3}_i = \alpha _0 + \alpha _1 \cdot \text {G1}_i + \alpha _2 \cdot \text {No-vaxxer}_i + \alpha _3 \cdot \text {Treatment}_i + \alpha _4 \cdot \text {Controls}_i + \varepsilon _i, \end{aligned}$$

where $\text {G3}_i$ is the guess of individual *i* in Game 3 about what is the fraction of the others that contributed in Game 2, $\text {G1}_i$ is the fraction of tokens invested by individual *i* in Game 1, $\text {No-vaxxer}_i$ is a binary variable with value 1 if individual *i* is a no-vaxxer and 0 otherwise, $\text {Treatment}_i$ is a categorical variable taking values in $\{T1, T2, T3\}$ depending on the treatment randomly assigned to individual *i*, $\text {Controls}_i$ includes *i*’s age and region of residence in Italy and $\varepsilon _i$ is an idiosyncratic error. The OLS results regression 1 is shown in Table 3a, where the coefficient corresponding to the dummy variable No-vaxxer indicates that being a no-vaxxer is associated with a reduction of 4.6%, in absolute terms, of the individual’s guess in Game 3 on the percentage of other subjects who will contribute, with a 5% significance level. In other words, no-vaxxers tend to believe that fewer other individuals will contribute to a public good.

Second, we assess the impact on the contribution to the public good in Game 2 with the following regression:

2 $$\begin{aligned} {\mathbb {P}}\left( \text {G2}_i = \text {Yes}\right) = \beta _0 + \beta _1 \cdot \text {G1}_i + \beta _2 \cdot \text {G3}_i + \beta _3 \cdot \text {No-vaxxer}_i + \beta _4 \cdot \text {Treatment}_i + \beta _5 \cdot \text {Controls}_i + \delta _i, \end{aligned}$$

where $\text {G2}_i$ is a binary variable indicating the contribution to the public good of individual *i* in Game 2 and $\delta _i$ is an idiosyncratic error. The results of a probit model are shown in Table 3b, together with an OLS model shown as a robustness check. Analyzing the marginal effects of G1 and G3 it can be seen that both are significant at a 1% level, while being a no-vaxxer is not significant. Together with the previous result of the OLS model in Table 3a, these findings suggest that the effect of being a no-vaxxer on the reduced contribution in Game 2 is mediated via G3. In other words, no-vaxxers believe that fewer others will contribute, and therefore they contribute less to the public good.

**Table 2:** Descriptive statistics of the variables considered in the statistical analysis.

Statistic	Mean	St. Dev.	Min	25-th *p*.	50-th *p*.	75-th *p*.	Max
G1	0.53	0.26	0	0.35	0.50	0.60	1
G2	0.72		0		1		1
G3	0.62	0.27	0	0.50	0.63	0.82	1
No-vaxxer $=$ True	0.12	0	0		0		1
Age	43	11.8	18	35	44	52	73

The number of observations (i.e., individuals) is 1482. In the 3 treatments T1, T2 and T3 there were 497, 512 and 473 individuals, respectively.

So, we find that in a public good game no-vaxxers contribute less, not because they are different in their level of risk aversion, but because they have less trust in others’ contribution. In the following we make use of a behavioral model to show that the two groups do not differ either in terms of the way they process their beliefs and take decisions.

**Table 3:** Results of the statistical analysis of the regression Eqs. 1 and 2.

	Dependent variable:	
	G3	
**(a)**		
G1	0.129$$^{***}$$	
	(0.027)	
No-vaxxer = True	−0.046$$^{**}$$	
	(0.022)	
Treatment = T3	0.065$$^{***}$$	
	(0.017)	
Treatment = T1	0.016	
	(0.017)	
Controls	Yes	
Constant	0.545$$^{***}$$	
	(0.058)	
Observations	1482	
$\hbox {R}^{2}$	0.042	
Adjusted $\hbox {R}^{2}$	0.026	
Residual Std. Error	0.270 (df = 1457)	
F Statistic	2.674$$^{***}$$ (df = 24; 1457)	

	Dependent variable:	
	G2	
	OLS	Probit
	(1)	(2)
**(b)**		
G1	0.119$$^{***}$$	0.126$$^{***}$$
	(0.039)	(0.039)
G3	0.799$$^{***}$$	0.690$$^{***}$$
	(0.037)	(0.028)
No-vaxxer = True	−0.033	−0.029
	(0.031)	(0.031)
Treatment = T3	0.131$$^{***}$$	0.126$$^{***}$$
	(0.025)	(0.022)
Treatment = T1	0.028	0.018
	(0.024)	(0.022)
Controls	Yes	Yes
Constant	0.151$^{*}$	
	(0.084)	
Observations	1482	1482
$\hbox {R}^{2}$	0.293	
Adjusted $\hbox {R}^{2}$	0.281	
Log Likelihood		−639.126
Pseudo $\hbox {R}^2$		0.271 (df = 26)
Akaike Inf. Crit.		1,330.252
Residual Std. Error	0.380 (df = 1456)	
F Statistic	24.130$$^{***}$$ (df = 25; 1456)	

$$^{*}p<$$0.1; $$^{**}p<$$0.05; $$^{***}p<$$0.01.

(a) OLS model of regression 1 of the form G3 $\sim$ G1 + No-vaxxer + Treatment + Controls. Controls include age and region. Standard errors in parenthesis.

(b) OLS and Probit model of regression 2 of the form: G2 $\sim$ G1 + G3 + Treatment + Controls. Coefficients of ordinary least square in column 1 and average marginal effects of Probit in column 2. Controls include age and region. Standard errors in parenthesis.

### A classification of subjects based on rationality and other-regarding preferences

We use the outcomes of Games 1 and 3, i.e., risk aversion elicitation and beliefs about others’ behavior, to statistically predict what our subjects will do in Game 2 and the actual contribution game as well as to theorize some assumptions about their preferences. To do so, we combine the outcomes of Games 1 and 3 to analyze, as a benchmark, what would be consistent to do for a purely selfish and perfectly rational *homo economicus*^20^.

As an example, suppose that a respondent plays $q = 100$ tokens (i.e., €1) in Game 1 and in Game 3 guesses that $t=20$ other players of her group had contributed in Game 2. Moreover, suppose that in Game 2 she was playing in T2. Then, for such a player, it would not be rational to contribute in Game 2 if she were selfish. However, if she had enough positive other-regarding preferences, then this could indeed make contributing in Game 2 rational. More precisely, with the numbers of this example, she would contribute if her preferences are such that she attributes a *monetary* value to the pleasure of contributing of at least €0.86. We call *C* this threshold value, and we classify subjects into three categories: (*i*) those who are consistent with *homo economicus*; (*ii*) those who are rational but have positive other-regarding preferences: they are willing to forego their own expected outcome to increase others’ expected outcome; and (*iii*) those who are rational but have negative other-regarding preferences: they are willing to forego their own expected outcome to decrease others’ expected outcome.

According to this classification: 968 respondents (65.3%) are of type (I), 66 (4.5%) are of type (II) and 448 (30.2%) are of type (III). When we separate vaxxers and no-vaxxers we obtain the following percentages: for type (I) 65.6% of vaxxers and 63.1% of no-vaxxers, for type (II) 4.4% of vaxxers and 5.0% of no-vaxxers, and for type (III) 30.0% of vaxxers and 31.8% of no-vaxxers. However, the $\chi ^2$ test shows that these differences are not statistically significant ($p=0.79$). To sum up, this classification seems to suggest that the majority of respondents are willing to forego their own outcome to benefit others’ outcomes (around 65% are of type (*ii*)), while around 30% of respondents behave as selfish agents (i.e., 30% are of type (*i*)). The motivations for such behaviors do not differ statistically between vaxxers and no-vaxxers, even if slightly more vaxxers are of type I. In the Supporting Information we describe in detail how we operate this classification. There, we also study the distribution of the abovementioned threshold *C* in the two populations of subjects, vaxxers and no-vaxxers, and perform additional checks to see whether they differ in the distribution of this parameter. Again, we found no statistically significant difference between the two groups in this respect.

**Table 4:** Classification of respondents based on rationality and other-regarding preferences.

	No-vaxxer = False (%)	No-vaxxer = True (%)	Total
Type (*i*)	30.0	31.8	448 (30.2%)
Type (*ii*)	65.6	63.1	968 (65.3%)
Type (*iii*)	4.4	5.0	66 (4.5%)
Total	12.1	87.9	1,482 (100%)

Type (*i*) individuals play the Games consistently with the assumptions of *homo economicus*. Type (*ii*) are rational and have positive other-regarding preferences. Type (*iii*) are rational but have negative other-regarding preferences. The $\chi ^2$ test shows that the differences between vaxxers and no-vaxxers are not statistically significant $(p = 0.79)$.

## Discussion

In the recent literature, several papers have highlighted the importance of using behavioral economics to guide policy actions because the design and effectiveness of a policy depend on the understanding of people’s incentives and beliefs^21–25^. In the case of policies aimed at maximizing the number of vaccinated people, it is crucial to understand the behavior and the reasoning of the so-called no-vaxxers, as opposed to the rest of the population that has already been vaccinated.

To the best of our knowledge, this is the first work where no-vaxxers are described in terms of incentive-based behavioral traits and where similarities and differences with the rest of the population are studied.

We show that no-vaxxers contribute less to simple public good experimental games where a certain fraction of the population needs to contribute for the public good to be effective. Since we also have information from the other two games, we can explain this difference with a statistical analysis of our data and with a theoretical framework. With the statistical analysis, we show that the difference in contribution is not due to a difference in risk aversion but to the fact that no-vaxxers have a lower level of trust that other people will also contribute to the public good. With the help of the theoretical model, we also show that vaxxers and no-vaxxers are not different in terms of rationality and other-regarding preferences. This suggests a causality relation because if we assume that they reason in a similar way and that they have similar incentives, then the only reason why they behave differently must be because they have different perceptions: no-vaxxers trust less in others, which explains their behavior in the public good game. We do not know if this is also the main reason why they show reluctance toward actual vaccination, but our results contribute to the analysis of this policy issue.

### Ethical approval and registration

Informed consent was obtained and this study was approved by the Ethics Committee for Research in the Human and Social Sciences (CAREUS) of the University of Siena (*Verbale CAREUS* dated May 13, 2021) and all research was performed in accordance with relevant guidelines and regulations. The trial number is AEARCTR-0008408 and the trial registration is available at this link: https://www.socialscienceregistry.org/trials/8408.

## Supplementary Information

Supplementary Information.

## Data Availability

The text of the survey and data of this study are available at the following GitHub link: https://github.com/alessiomuscillo/no_vax_and_games.
