# Supplementary material for: No-vaxxers are different in public good games
Source: Sci Rep. 2022 Oct 28;12:18132. doi: 10.1038/s41598-022-22390-y (PMC9616914; doi:10.1038/s41598-022-22390-y)
Supplement: Supplementary file 1 — Supplementary Information. [file 41598_2022_22390_MOESM1_ESM.pdf]

Supporting Information for:

No-vaxxers are different in public good games

Marcello Basili[1], **Alessio Muscillo**[1,*], **and Paolo Pin**[1,2]

[1]Università di Siena, Department of Economics and Statistics, Siena, 53100, Italy
[2]Università Bocconi, BIDSA, Milan, 20136, Italy
[*]corresponding author: alessio.muscillo2@unisi.it

ABSTRACT

This file is an appendix to the main article titled "No-vaxxers are different in public good games" by M. Basili, A. Muscillo and P. Pin. In this file, in Section 1 we report chronologically the impact of the Covid19 pandemic on Italy, to provide a picture of the period in which we conducted the survey, then in Section 2 we describe the survey in detail and in Section 3 we explain and analyze the data more in depth and the analysis that we have conducted. Lastly, Section 4 contains additional figures.

1 Extended Introduction on the Covid19 Pandemic with a Focus on Italy

The new coronavirus that has been spreading around the world causes a respiratory illness that can be severe. In early December 2019 unknown severe pneumonia cases appeared in Wuhan, capital of the Hubei province, and rapidly spread throughout China. Michael Worobey, a leading expert in tracing the evolution of viruses at the University of Arizona, suggests that the first known patient sickened with the coronavirus was a vendor in the Huanan Seafood Wholesale Market, where in the end of December 2019, doctors at several Wuhan hospitals noticed mysterious cases of pneumonia arising in people who worked at the Huanan Seafood Wholesale Market. The first official public report issued by the Wuhan Municipal Health Commission reported that 27 patients were related to the Huanan market and all of them were transferred to Jinyintan Hospital, Wuhan's premier infectious disease center, from 29 December to 2 January[1].

In 29 December 2019, the new coronavirus disease was identified as a severe acute respiratory syndrome and called SARS-CoV-2, because of genetic similarity to SARS-CoV, the Severe Acute Respiratory Syndrome appeared in 2003 (8.096 cases and 774 deaths). SARS-CoV-2 is also similar to MERS-CoV (Middle East Respiratory Syndrome coronavirus) first reported in Saudi Arabia in 2012 and spreaded to 27 countries with 858 deaths. Coronaviruses are naturally hosted in animals and are able to infect humans (species leap). SARS-CoV-2, SARS and MERS are included in the large family of *Coronaviridae* and have genetic similarity from 79% (SARS-CoV-2 and SARS) to 50% (SARS-CoV-2 and MERS).

As of December 2021, all around the world more than 256 million of people got Covid-19, 233 million were hospitalised, more than 19 million are active cases with 80,000 critical condition, and 5,2 million of deaths, so that the case fatality rate is 2,2%. Nevertheless, many scientists and experts consider such data underestimated by at least 35%, because many countries undercounted the number of fatalities, in fact by comparing the number of excess deaths with baseline it is observed that this excess is greater than the number of Covid-19 fatalities officially recorded by the government. Crucially, Islam and colleagues set "an estimated 979 000 (95% confidence interval 954 000 to 1 001 000) excess deaths occurred in 2020 in the 29 high income countries analysed"[2].

At the same time a total of 7.37 billion of vaccine doses have been administrated and 4.18 billion people received one dose. Crucially, there is a stark gap in vaccination all around the world: 75% of doses have been administered in developed countries, but only 0.7% of doses have been administered in low-income countries, so that just 9.7% of the population receiving at least one dose of a vaccine, in Africa. It is the failure of vaccine-sharing program called Covax which aimed to provide at least 2 billion of doses in current year but it is expected to have at more 1,4 billion of doses available at the end of 2021.

Effects of vaccination on Covid-19 diffusion and consequences are effective and huge: vaccination reduces the risk of dying or being hospitalised with Covid-19 of 90%, in a French SNDS (Système National des Données de Santé) study of 22.6 million people over the age of 50, and a protection against death of 72% in Israel[3].

By the end of Autumn 2021, a fourth wave of pandemic has loomed all around the world and violent anti-vaxxers, so called no-vax people, have started protesting against mass vaccination. We think that there is more than a correlation effect between existence of a relatively large movement of no-vaxxers (10-15% of population) and the new wave of Covid-19. Long term prospects for pandemic assume that Covid-19 could become an endemic disease such as an very bad influenza. In such a case it is expected at least four years of successive waves for the pandemic disease: the Spanish flu (AH1N1) occurred in four waves

from 1918 to 1920 and infected one-third of the world's population (at least 500 million) and killed from 19 million to 50 million of people[4] (CDC pandemic Influenza), with a mortality rate between 1% to 3% and a case fatality ratio between 4% to 10%. There is a crucial difference between Spanish flu and Covid-19, the former induced large mortality in younger people than older ones, the latter the opposite.

Covid-19 is a new disease and human beings have few experience with similar diseases (SARS and MERS), that never evolved in pandemic and forecasts about this disease are very uncertain and not fully reliable. It was firstly supposed that mass vaccination would eventually induce immunity to SARS-CoV-2 to block most transmission and produce the so called 'herd-immunity'. Herd immunity is obtained when immunized people reach a given threshold that is determined by a simple equation: $\mathrm{HI} = 1 - 1/R_0$, where R_0 is the basic reproduction number of the disease. Crucially, R_0 of Covid-19 was estimated between 3.19-5.24 of infected cases of CoVid-19 and between 2.53-6.72 from sensitivity analysis (WHO). Unfortunately, herd immunity appears to be difficult to reach for several reasons: the threshold seems higher than the initially estimated 70% of vaccinated and past exposed to the virus people, corresponding to $R_0 \approx 3$; vaccines have still unclear effects on blocking the virus transmission; mass vaccination has been delayed and has been done unevenly across the world with the emergence of geographic clusters. Last but not least, new variants can be more transmissible and resistant to vaccines.

In such an uncertain scenario, in which immunity could not last forever and non-pharmaceutical measures, such as facial mask and social distancing, could be essential to keeping cases down, many people all around the world, the so-called no-vaxxers, reject vaccination and any suggested precautionary measures. Often no-vaxxers not only think that vaccines are not effective and are not able to reduce the transmission of the virus, but also that vaccines could not prevent severe disease, hospitalizations, admission to intensive care unit (ICU) and deaths. Moreover, no-vaxxers show 'vaccine hesitancy' and state that vaccines are not safe and could induce serious types of health problems after vaccination, such as anaphylaxis and thrombosis with thrombocytopenia syndrome (TTS) and/or unknown long-term side effects. Crucially, anaphylaxis and TTS are very rare and long term health are very unlikely, since "vaccine monitoring has historically shown that side effects generally happen within six weeks of receiving a vaccine dose".[1]

In a retrospective surveillance study, Haas and colleagues[5] "estimated that Israel's vaccination campaign averted 158 665 (95% CI 144 640–172 690) SARS-CoV-2 infections, 24 597 (18 942–30 252) hospitalisations, 17 432 (12 770–22 094) severe or critical hospitalisations, and 5532 (3085–7982) deaths [...]. Without the national vaccination campaign, Israel probably would have had triple the number of hospitalisations and deaths compared with what actually occurred during its largest wave of the pandemic to date, and the health-care system might have become overwhelmed".

In high-income countries, scientists set a framework called the 5C model to scrutinize 'vaccine hesitancy' and elicited five main individual determinants for vaccine hesitancy: confidence, complacency, convenience (or constraints), risk calculation, and collective responsibility. Machingaidze and colleagues[6] in an analysis including 15 studies carried out in Africa, South Asia, Latin America, Russia and the United States show that "[t]he data show that vaccine acceptance is explained mainly by an interest in personal protection against COVID-19, whereas concerns about side effects are the most common reasons for hesitancy, and health workers are the most trusted sources of guidance about vaccines against COVID-19". Crucially they found that "With the wide availability of smartphones, more people can now access the internet and social media in LMICs. Although this can be a great tool for self-education, which is a key component of vaccination decision-making, it also presents several challenges in the form of misinformation (including 'anti-vaxx' messaging) and incomplete information, as well as inconsistent and complicated scientific information that may be difficult to understand"[6]. When comparing a cohort of 6.4 million COVID-19 vaccines and 4.6 million demographically similar unvaccinated persons and calculating standardized mortality rates (SMRs) or deaths per 100 person-years and compared with a rate ratio test between vaccinated and unvaccinated groups, it was found that "SMR was 0.34-0.35 for vaccinated people and 1.11-1.47 for unvaccinated people"[7].

About side effects of vaccination, many studies set that short-term adverse effects are moderate in frequency, mild in severity, and short-lived. Side effects are more frequent in younger individuals, women, and among previously infected people.

Italy was hit by the CoVid-19 outbreak in mid February, but very likely the virus was spreading in some Regions since January: Lombardy, Veneto and Emilia-Romagna (so-called new industrial triangle). Occurrence of the epidemic in North Italy does not appear by chance but it has been envisaged as the possible consequence of the commercial interconnections and relationships (supply-chain) between those Regions with China. The same destiny hit industrial regions (landers) in Germany, i.e. Bavaria, and France. In the new Italian industrial triangle the exports/GDP rate is 40% similar to some German landers one. This common aspect shed a light about the disease carriers: businessman were the modern sailors and traders, who spanned the CoVid-19 disease, not tourists. Crucially they violated the travel ban by triangulation with other countries and spread the CoVid-19 in Northern Italy.

On 21 December 2020, the European Medicines Agency (EMA) recommended the first COVID-19 vaccine. As other European countries, Italy launched its COVID-19 vaccination campaign with four vaccines on 27 December 2020. Currently, in Italy 93,4 million of doses have been administrated, 44 million of people received full vaccination (73,9%) and more than 664

[1] https://www.cdc.gov/coronavirus/2019-ncov/vaccines/safety/safety-of-vaccines.html

thousands received the third dose or buster.

Given more than 4.92 million of cases and 133 thousands confirmed deaths, Italian deaths for million are 2,288 and, unfortunately, the Italian case fatality rate is 2,79, the highest in the world.

Italian ISS (Istituto Superiore di Sanità) analyzed over 29 million of people (more than 56% of the Italian population aged ≥16 years) who received at least one dose of an mRNA COVID-19 vaccine by mid-August and followed-up to the 29th of August 2021. ISS found that "the incidence of any COVID-19 diagnosis declines from 1.13 per 10,000 person-days in the first 14 days after the first dose, to 0.34 after the second dose ...Similar estimates are obtained when considering COVID-19 diagnoses with subsequent hospitalization (overall: 0.17 and 0.02 per 10,000 person-days, respectively in the first 14 days after the first dose and after the second dose, admission to ICU (0.02 to 0.001, or death within 28 days from diagnosis (0.05 to 0.01, Table 4), also when stratifying for the characteristics here considered ... The incidence of any COVID-19 diagnosis declines from 1.13 per 10,000 person-days in the first 14 days after the first dose, to 0.34 after the second dose" (ISS Report n.4, 30/09/2021).

The Italian Medicines Agency[8] reports 120 communicated side effects per 100.000 doses administrated for all vaccines, indeed 101,110 per 84,010,605 doses administrated (53,43% first dose; 46,51% second dose and 0,06% third dose) respectively 71% female and 28% male; only 14,4% were serious side effects (17 per 100.000 doses administrated) that resolved completely or partially positive way. In mixed vaccinations adverse reaction are 40 per 100.000 doses administrated. Crucially deaths were 0,72 per 100.000 doses administrated (608 deaths, respectively 397 after the first dose and 211 after the second dose) which 50,8% female and 48,2 male 76 years old (average). In detail: Caminarty 391 deaths (0.65 per 100,000), Spikevax 96 (0.91 per 100,000), Vexzeria 98 (0.81 per 100,000) and Jansen 23 (1.56 per 100,000). Critically, 435/608 deaths (71.5%) shown a positive evaluation for causality with respect to the OMS's algorithm, but about it 259/435 cases (59,5%) are not correlated with vaccination, 133/435 are indeterminate and 27/435 (6.2%) unclassified due to incomplete information. Only 16 out of 435 cases (3.7%) are correlated with administered doses.

2 The Survey

2.1 Ethical Approval and Registration

The survey was implemented in Qualtrics and conducted on a representative sample of the Italian population in September 2021. Informed consent was obtained and this study was approved by the Ethics Committee for Research in the Human and Social Sciences (CAREUS) of the University of Siena (*Verbale CAREUS* dated May 13, 2021) and all research was performed in accordance with relevant guidelines and regulations. The trial number is AEARCTR-0008408 and the trial registration is available at this link:
https://www.socialscienceregistry.org/trials/8408.

2.2 Detailed Description of the Survey

The survey consists of 3 games and then it is concluded by a series of Covid-related questions. The games are incentivized so, in addition to a show-up fee, respondents are paid on the basis of the outcome they obtain in the games played. (They play the 3 games, then one of them is randomly drawn and they obtain what they got in that game.) In the next section, we show the exact text of the survey. Here we summarize the survey flow (see Figure SI.1), which is as follows.

- In the beginning, respondents face a comprehension test to check their level of attention: only those who answer correctly are allowed to continue the survey, so we end up with 1,482 individuals. In Section 3.1 we show that those who are left out are not statistically different from those included in the sample.
- Then, respondents play Game 1, Game 2 and Game 3. While Game 1 is the same for everyone, Game 2 and Game 3 have different versions that depend on the treatments (more details below).
- Lastly, respondents are asked Covid- and vaccine-related questions.

The games are as follows.

(G1) Game 1 is a risk elicitation game,[9] where respondents are given 200 tokens and asked the amount $q \in [0,200]$ they want to invest in a non-repayable project that succeeds with probability 50% and yields $2.5q$. The respondent keeps what is not invested, i.e. $200-q$.

(G2) Game 2 is a (one-shot binary threshold) public game,[10] where respondents are said that they are grouped with other respondents and that they have the possibility of participating or not in a project to produce a good that will benefit everyone, including those who did not participate. The project will be executed only if the number of those who choose to participate meet or exceed a known threshold. Otherwise, the project is not executed and the public good is not produced.

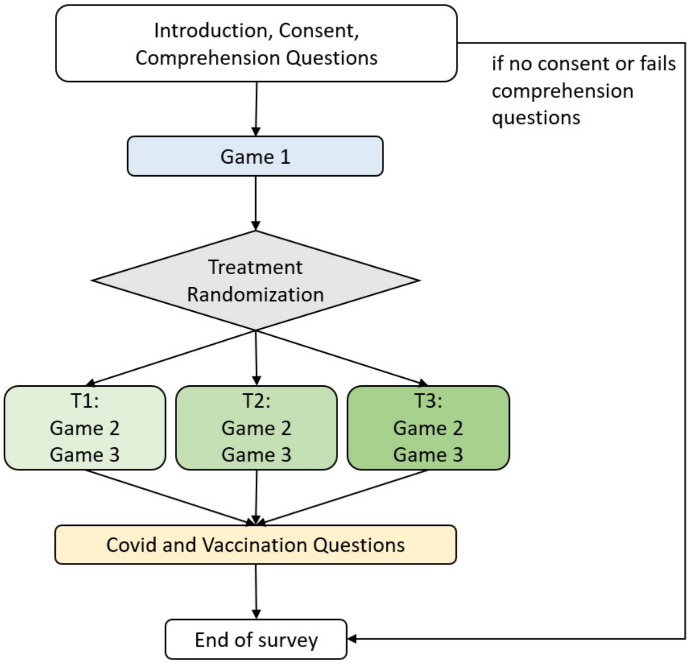

Figure SI.1. Survey flow. After introduction and comprehension questions, the survey proceeds with Game 1, which is a risk elicitation game. Then individuals are randomly assigned to one of three treatment T1, T2 or T3 all consisting of a variation of a public good game (Game 2) and a belief elicitation game about the others' behavior (Game 3). The survey ends with Covid- and vaccination-related questions.

Individuals have the incentive to free ride on the others' participation to the project. The respondent's actions and payoffs are summarized below.

(G3) Game 3 is a game of belief elicitation, where every respondent is asked how many in her group she thinks had participated in Game 2. The payoffs are described below.

After Game 1 individuals are evenly and randomly assigned to one of the following treatments.

(T1) Treatment 1, also called 'small and risky', is such that in Game 2 the groups are of 5 people and the threshold to exceed to produce the public good is 3 out of 5 individuals. Moreover, if the threshold is not met nor exceeded, those who decided to participate in the project lose their investment.

(T2) Treatment 2, also called 'large and risky' is like T1 with the exception that now the groups are of 50 people and the threshold is of 25 individuals.

(T3) Treatment 3, also called 'large and safe', is such that groups are of 50 people and the threshold is 25 individuals, as in T2. However, in this treatment if the threshold is not met nor exceeded, those who decided to participate get an individual benefit (instead of losing their investment, as in the 'risky' cases of T1 and T2), so that participating in Game 2 is a 'safer' choice with respect to T1 and T2.

The payoffs of Game 2 and Game 3, which depend on the treatment, are as follows:

- in Game 2:
  - for T1 and T2:
    - ∗ if the respondent contributes and the threshold is not reached, then she gets 0 tokens;
    - ∗ if the respondent contributes and the threshold is reached or exceeded, then she gets 300 tokens;
    - ∗ if the respondent does not contribute and the threshold is not reached, then she gets 100 tokens;

    * if the respondent does not contribute and the threshold is reached or exceeded, then she gets 400 tokens;
  - for T3:
    * if the respondent contributes and the threshold is not reached, then she gets 200 tokens (this is the only case that differs from T1 and T2);
    * if the respondent contributes and the threshold is reached or exceeded, then she gets 300 tokens;
    * if the respondent does not contribute and the threshold is not reached, then she gets 100 tokens;
    * if the respondent does not contribute and the threshold is reached or exceeded, then she gets 400 tokens;
- in Game 3:
  - for T1:
    * if the respondent's guess is exactly equal to the number of others' in her group who have contributed, she gets 300 tokens;
    * if the difference between the respondent's guess and the number of others' in her group who have contributed is of 1, then she gets 150 tokens;
    * if the difference between the respondent's guess and the number of others' in her group who have contributed is of 2, then she gets 100 tokens;
    * if the difference is greater, she gets 50 tokens;
  - for T2 and T3:
    * if the respondent's guess is exactly equal to the number of others' in her group who have contributed, she gets 300 tokens;
    * if the difference between the respondent's guess and the number of others' in her group who have contributed is of 2, then she gets 150 tokens;
    * if the difference between the respondent's guess and the number of others' in her group who have contributed is of 10, then she gets 100 tokens;
    * if the difference is greater, she gets 50 tokens;

After the games are played, respondents are asked a series of Covid19- and vaccination-related questions. Crucially, the games are not framed in a way that can recall or refer to the pandemic situation, Covid19, vaccines or public goods. In particular, we ask whether they are vaccinated or not and, if not, whether they do not want to get vaccinated or they were not able to get vaccinated but they want to get vaccinated as soon as possible. We then categorize as *no-vaxxer* those respondents who are not vaccinated and say that do not want to get vaccinated and they amount to around 12% of the sample. Notice that those who are not vaccinated are around 15% of the sample. These figures are in line with the estimation provided by the Italian government of 7.5 million of people not vaccinated in Italy at the time of the survey.[11]

2.3 Survey Text

The survey was only intended for Italian respondents. The text of the survey (in Italian) and its English translation are available at the following link: https://github.com/alessiomuscillo/no_vax_and_games

3 More Details on Data

In this section we show additional information and descriptive statistics about the respondents. In Subsection 3.1 we show that the individuals who consent to participate to the 3 games and pass the comprehension questions are not statistically different from those who do not. In Subsection 3.2 we show additional information and comparisons on individuals who are vaccinated for Covid and those who are not. Lastly, in Subsection 3.3 we show a model to classify subjects on the basis of rationality and other-regarding preferences.

3.1 Comparison of individuals in sample and excluded from sample

In this section we describe the difference between the individuals who are in the sample with those out of the sample (because they did not consent to participate to the experiment or did not pass the comprehension questions). We compare their age (Figure SI.4a), region where they reside (Figure SI.4b) and device used (Figure SI.4c. Naturally, the duration of the survey is affected by not participating (Figure SI.4d) because, if consent is not given or comprehension questions are not passed, the survey ends automatically (thus shortening the time).

Out of 2,546 individuals who took part to the survey, those who did not give consent to participate or failed to pass the comprehension questions are 1,064 people. The χ^2 tests show that there is no strong statistical difference between the two samples in terms of age ($p = 0.062$), region ($p = 0.7$) and device used ($p = 0.277$), thus showing that those who participated to the survey do not different significantly from those who did not.

3.2 Additional information on vaxxers and no-vaxxers

In the paper we show that in Game 2 no-vaxxers contribute significantly less to the public good than the others (Barnard test $p = 0.048$). When we separate by treatment (Figure SI.5) we obtain that this result is no longer significant:

- for T1: Barnard $p = 0.381$;
- for T2: Barnard $p = 0.331$;
- for T3: Barnard $p = 0.274$.

Concerning Game 3, when respondents guess how many of the others they think have contributed in Game 2, we classify the respondents in:

- *pivotal* if their guess was $T-1$, so that they think that their contribution is what is missing in order to produce the public good;
- *below threshold* if their guess was below $T-1$, so that their contribution was not needed for the production of the public good and the public good was not produced anyway;
- *above threshold* if their guess was above T, so that their contribution was not needed for the production of the public good and the public good was produced anyway.

With this classification, in Game 3 (Figure SI.6) there is no significant difference between the guesses of vaxxers and no-vaxxers (χ^2 test $p = 0.195$). When separated by treatment (Figure SI.7), the result shows significant difference for T2 (χ^2 test $p = 0.044$) but remains non-significant for T1 and T3 (respectively, χ^2 test $p = 0.269$ and $p = 0.852$).

As last question, we asked respondents' feeling about how the evolution of the pandemic situation in the short term (next 2 months) and in the long term (next 2 years). We then classify respondents in *optimist* and *pessimist* according to their answer (e.g., "Covid will no longer be dangerous" or "Covid will continue to be dangerous"). Interestingly, in the short term no-vaxxers are significantly more optimistic (Barnard test $p \approx 0$, see Figure SI.8a) while in the longer term the difference in optimism is reduced and, additionally, becomes statistically non-significant (Barnard $p = 0.138$, see Figure SI.8b).

3.3 A classification of subjects based on rationality and other-regarding preferences

Let us analyze Game 1. Each respondent chooses $q \in [0,200]$ tokens (with 1 token corresponding to 0.01 Euros) to invest in the risky lottery with an expected utility of

$$\frac{1}{2} \cdot U(200-q) + \frac{1}{2} \cdot U\left((200-q) + \frac{5}{2}q\right) = \frac{1}{2} \cdot U(200-q) + \frac{1}{2} \cdot U\left(200 + \frac{3}{2}q\right).$$

and if she acts optimally, then the optimal choice q satisfies first order conditions

$$U'(200-q) = \frac{3}{2} \cdot U'\left(200 + \frac{3}{2}q\right). \tag{1}$$

To infer risk aversion from small lotteries, it is reasonable to assume a constant absolute risk aversion (CARA) utility of the form $U(x) = 1 - e^{-\lambda x}$, such that $U'(x) = \lambda e^{-\lambda x}$, then equation 1 becomes $\lambda \exp[-\lambda(2-q)] = \frac{3}{2}\lambda \exp\left[-\lambda\left(2+\frac{3}{2}q\right)\right]$ which gives

$$\lambda = \frac{2}{5q} \log\left(\frac{3}{2}\right) \simeq \frac{0.162}{q}. \tag{2}$$

Notice that $\lambda > 0$ always and $\lambda \to +\infty$ for $q \to 0^+$.

Notice, instead, that when assuming a constant relative risk aversion (CRRA) utility of the form $U(x) = \frac{x^{1-\lambda}}{1-\lambda}$, such that $U'(x) = x^{-\lambda}$, equation 1 becomes $(2-q)^{-\lambda} = \frac{3}{2}\left(2+\frac{3}{2}q\right)^{-\lambda}$ which gives $\lambda = \frac{\log(3/2)}{\log(4+3q)-\log(4-2q)}$ and notice that $4-2q > 0$ and $\lambda(q) > 0$, for $q \in (0,2]$. Moreover, $\lambda \to 0^+$ for $q \to 2^-$.

In Game 2, let p be the belief that a respondent has about the i.i.d. probability that any other player will contribute, with a Game 2 with N players and threshold T. In other words, $p \approx \frac{t}{N-1}$, where t is the guess made in Game 3.

Then the probability that exactly $T-1$ of the other $N-1$ players will contribute (i.e., the probability of being pivotal) is given by $\Delta_T := \binom{N-1}{T-1} p^{T-1}(1-p)^{N-T}$ and the probability that at least T and at least $T-1$ of the other $N-1$ players will contribute is, respectively, $P_T = \sum_{t=T}^{N-1} \binom{N-1}{t} p^t (1-p)^{N-1-t}$ and $P_{T-1} = \sum_{t=T-1}^{N-1} \binom{N-1}{t} p^t (1-p)^{N-1-t} \equiv P_T + \Delta_T$. Notice that if the guess in Game 3 is $t = 0$, then $p = 0$ and we can also say that the probability of being pivotal has to be $\Delta_T = 0$ and that also the probability that at least T or $T-1$ contributed have to be $P_T = P_{T-1} = 0$. On the other hand, if $t = N-1$ then the probability of being pivotal is $\Delta_T = 0$ but $P_T = P_{T-1} = 1$.

Now, let $R \in \{0,2\}$ be the contributing premium (depending of the treatment being "risky", i.e. T1 and T2, or "safe", i.e. T3) and let $C \in \mathbb{R}$ be a parameter capturing the satisfaction of a respondent for contributing to a successful public good measured in monetary terms, derived from altruistic intentions or from self-esteem considerations.

The respondent's expected utility from contributing and not contributing in Game 2 are then, respectively, $\Pi_c = (1-P_{T-1}) \cdot U(R+C) + P_{T-1} \cdot U(300+C)$ and $\Pi_{nc} = (1-P_T) \cdot U(100) + P_T \cdot U(400)$. Given a treatment (i.e., given R), for such a respondent (i.e., given q, p and C), it is rational to contribute in Game 2 if and only if

$$\Pi_c \geq \Pi_{nc}. \tag{3}$$

On the other hand, given how a respondent has played the games and assuming that she is rational, then we can infer the level C such that $\Pi_c \geq \Pi_{nc}$. Such C is the minimum level of other-regarding preference that makes contributing rational.

As an example, suppose that a respondent plays $q = 100$ tokens (i.e. €1) in Game 1 and in Game 3 guesses that $t = 20$ other players are contributing. If in Game 2 she was playing in T2, then for her it is rational to contribute only if $C > 86$ tokens (i.e.,). In other words, by construction the solution C to equation 3 is the minimum monetary value that makes it rational to contribute in Game 2. This means that if a respondent does not contribute, then her level of altruism cannot exceed the corresponding threshold C. Instead, if a player contributes then her level of altruism is at least C. This also implies that non-contributors should have lower Cs than contributors and, indeed, in the data we found that this difference is statistically significant (Figure SI.2a). We can also use the parameter C to check whether there is a difference between vaxxers and no-vaxxers. In the data, we found that this difference is not statistically significant (Figure SI.2b). This results are robust to the utility function used: in Figure SI.3 we show the same computations when a CRRA utility function is used instead of a CARA utility function.

Lastly, we can introduce the following classification if, for every respondent, we take the C that solves equation 3 and also take her response to Game 2:

(i) if $C \geq 0$ and $G2 =$ No or if $C \leq 0$ and $G2 =$ Yes, then the respondent is consistent with *homo economicus*'s behavior, that is, standard economic rationality and selfishness;

(ii) if $C \geq 0$ and $G2 =$ Yes, then the respondent has positive other-regarding preferences, as if she is willing forego own expected outcome to increase others' expected outcome;

(iii) if $C \leq 0$ and $G2 =$ No, then the respondent has negative other-regarding preferences, as if they are willing to forego own expected outcome to decrease others' expected outcome.

According to this classification: 968 respondents (65.3%) are of type (ii), 66 (4.5%) are of type (iii) and 448 (30.2%) are of type (i). When we separate vaxxers from no-vaxxers we obtain the following percentages: for type (ii) 65.6% of vaxxers and 63.1% of no-vaxxers, for type (iii) 4.4% of vaxxers and 5.0% of no-vaxxers, and for type (i) 30.0% of vaxxers and 31.8% of no-vaxxers. However, the χ^2 test shows that these differences are not statistically significant ($p = 0.79$). To sum up, this classification seems to suggest that the majority of respondents are willing to forego own outcome to benefit others' outcome (around 65%, of type (ii)), while around 30% of respondents behave as selfish agents (30%, of type (ii)). The motivations for such behaviors do not differ between vaxxers and no-vaxxers, even if it seems that slightly more vaxxers are of type (ii).

4 Additional Figures

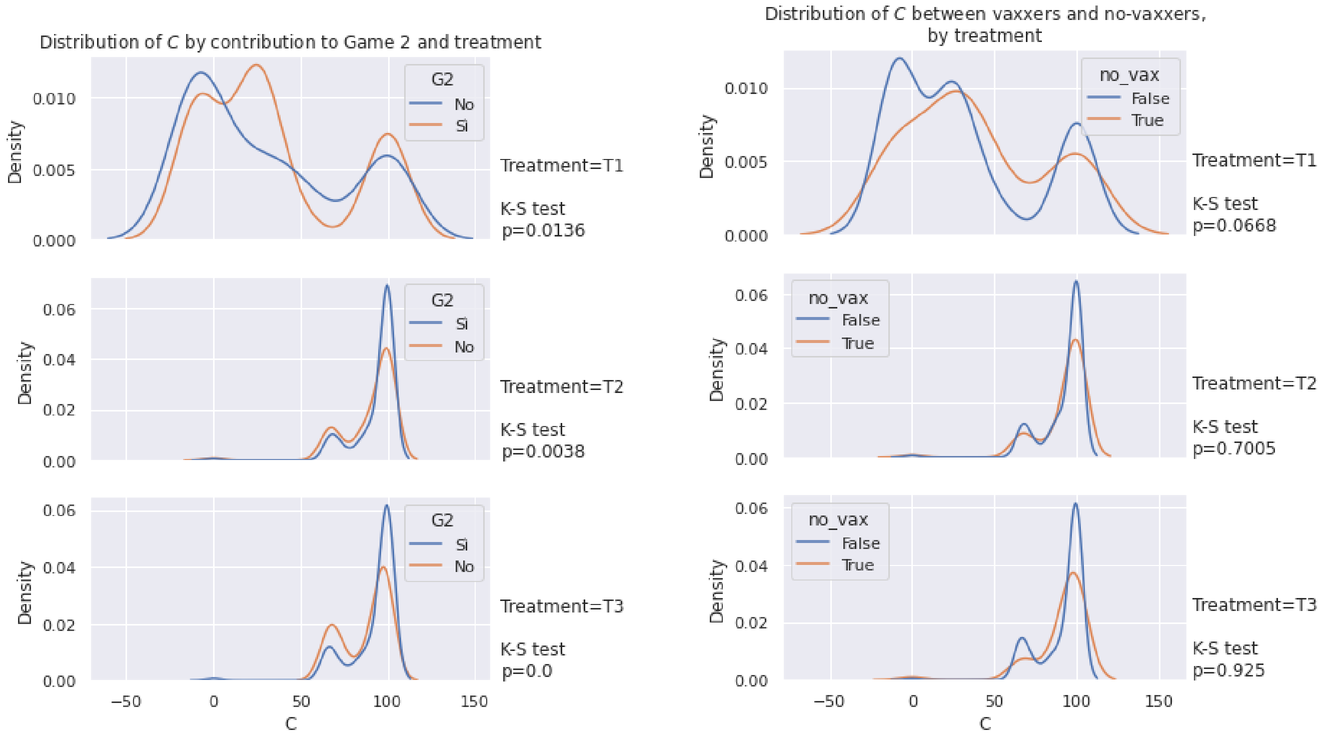

(a) *C* and Game 2.

(b) *C* and no-vaxxers.

Figure SI.2. Distribution of parameter *C*, assuming a CARA utility. P-values of the Kolmogorov–Smirnov test are reported.

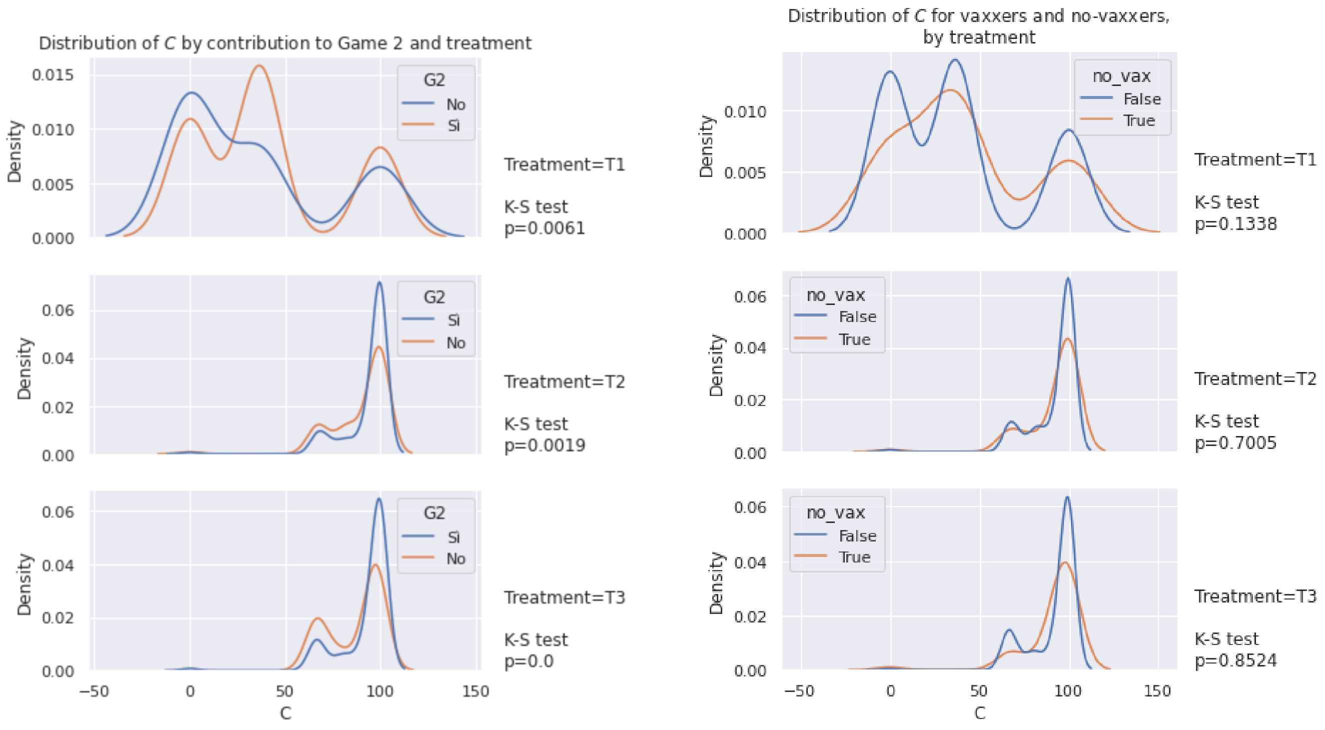

(a) *C* and Game 2.

(b) *C* and no-vaxxers.

Figure SI.3. Distribution of parameter *C* computed assuming a CRRA utility. P-values of the Kolmogorov–Smirnov test are reported.

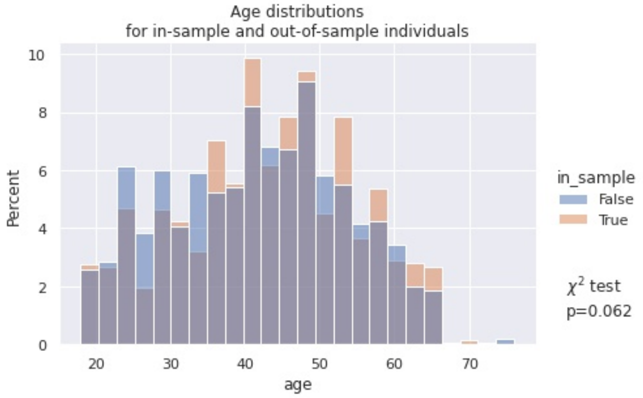

(a) Distribution of age of respondents for individuals in sample and out of the sample.

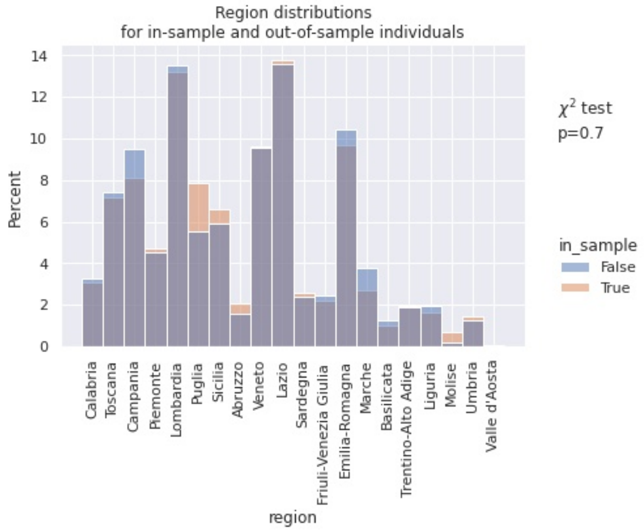

(b) Distribution of region of residence, for individuals in sample and out of the sample.

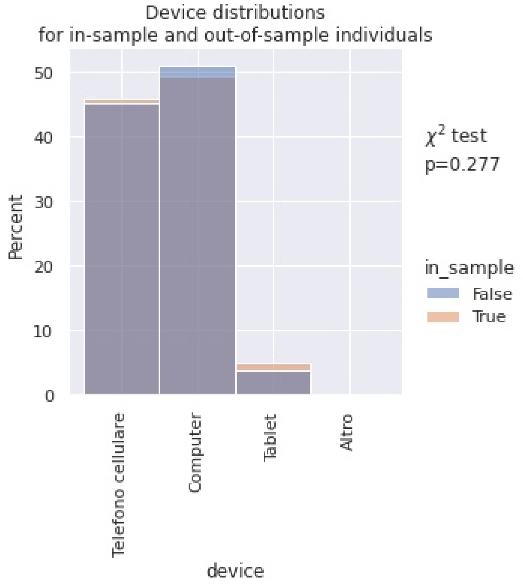

(c) Distribution of device used to do the survey, for individuals in sample and out of the sample.

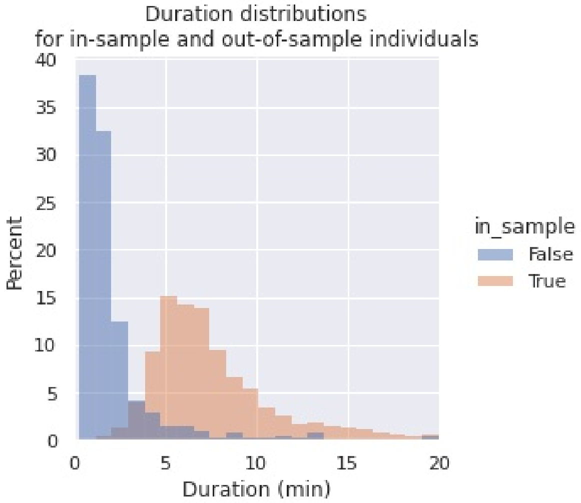

(d) Distribution of duration to perform the survey, for individuals in sample and out of the sample.

Figure SI.4. Distribution of age, region of residence, device used and duration, across individuals in sample and out-of-sample. In order to be in the sample, at the beginning of the survey each subject has to agree to the terms, give the correct answers to 2 comprehension questions and, lastly, agree to participate to the games.

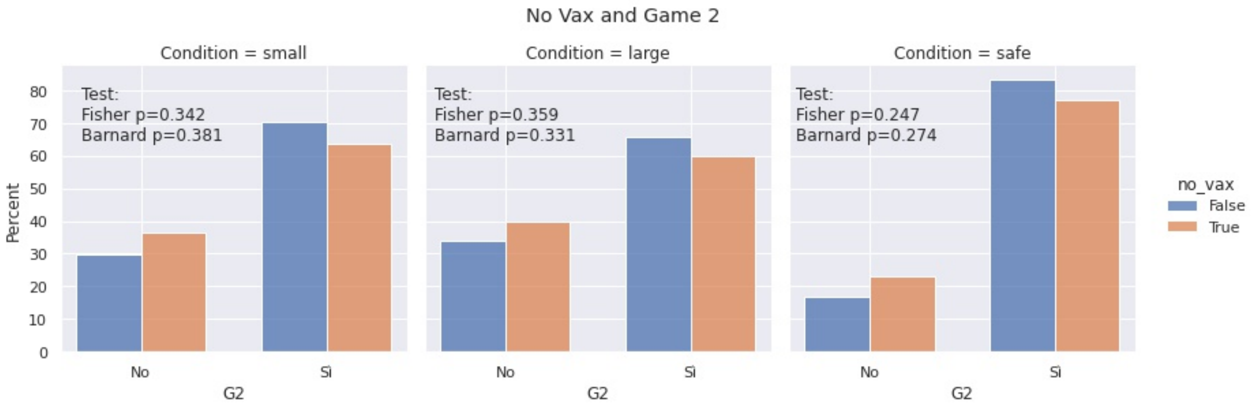

Figure SI.5. Contribution to the public good in Game 2, separated by treatment (a.k.a. Condition).

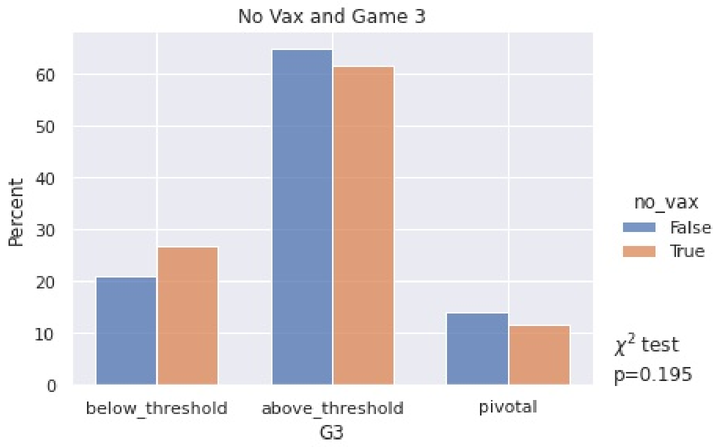

Figure SI.6. Game 3: Belief about others' behavior in Game 2. If the threshold in game 2 is T and the subject guesses that $t < T-1$ of the others will contribute, then she is classified as "below threshold". If the subject's guess is $t > T-1$, then she is classified as "above threshold". Lastly, if the subject's guess is $t = T-1$, then she is classified as "pivotal".

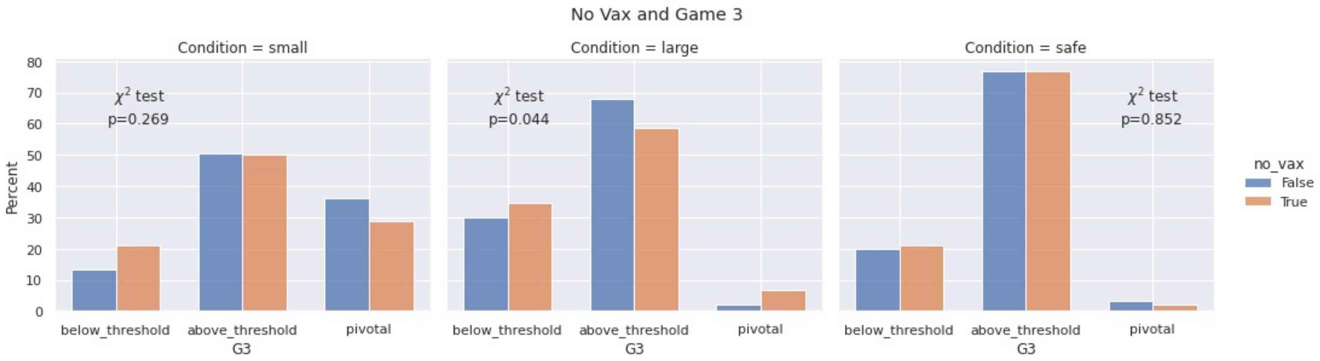

Figure SI.7. Game 3: Belief about others' behavior in Game 2, separated by treatment (a.k.a. Condition).

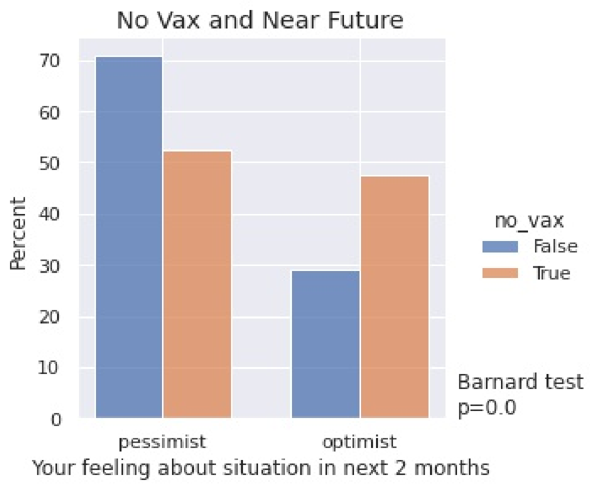

(a) “What is your feeling about the evolution of the pandemic situation in the next 2 months?”

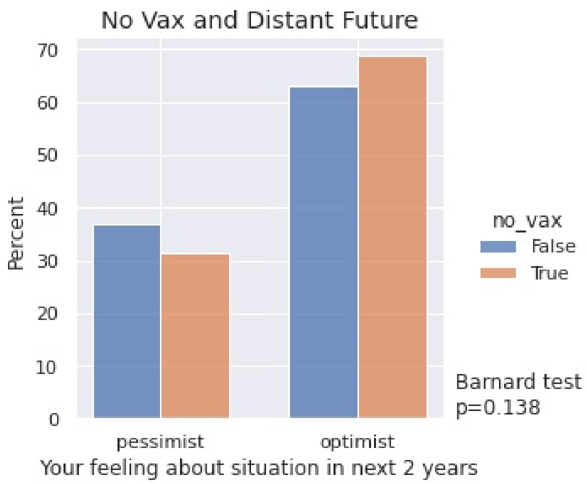

(b) “What is your feeling about the evolution of the pandemic situation in the next 2 years?”

Figure SI.8. Percentages of individuals classified as “pessimist” or “optimist” among no-vaxxers and vaxxers.

References

1. Worobey, M. Dissecting the early COVID-19 cases in Wuhan. *Science* **0**, eabm4454, DOI: 10.1126/science.abm4454 (2021).
2. Islam, N. *et al.* Excess deaths associated with Covid-19 pandemic in 2020: age and sex disaggregated time series analysis in 29 high income countries. *The BMJ* **373** (2021).
3. Jabłońska, K., Aballéa, S. & Toumi, M. The real-life impact of vaccination on COVID-19 mortality in Europe and Israel. *Public Heal.* **198**, 230–237, DOI: 10.1016/j.puhe.2021.07.037 (2021).
4. Spreeuwenberg, P., Kroneman, M. & Paget, J. Reassessing the global mortality burden of the 1918 influenza pandemic. *Am. J. Epidemiol.* **187**, 2561–2567 (2018).
5. Haas, E. J. *et al.* Infections, hospitalisations, and deaths averted via a nationwide vaccination campaign using the Pfizer–BioNTech BNT162b2 mRNA COVID-19 vaccine in Israel: a retrospective surveillance study. *The Lancet Infectious Diseases* **22**, 357–366 (2022).
6. Machingaidze, S. & Wiysonge, C. S. Understanding COVID-19 vaccine hesitancy. *Nat. Medicine* **27**, 1338–1339 (2021).
7. Xu, S. *et al.* COVID-19 Vaccination and Non–COVID-19 Mortality Risk – Seven Integrated Health Care Organizations, United States, December 14, 2020–July 31, 2021. Tech. Rep., Morbidity and Mortality Weekly Report 2021 (2021).
8. Rapporto sulla Sorveglianza dei vaccini COVID-19. https://www.aifa.gov.it/documents/20142/1315190/Rapporto_sorveglianza_vaccini_COVID-19_9.pdf (2021).
9. Gneezy, U. & Potters, J. An experiment on risk taking and evaluation periods. *The Q. J. Econ.* **112**, 631–645 (1997).
10. Diekmann, A. Volunteer's dilemma. *J. Confl. Resolut.* **29**, 605–610 (1985).
11. Report settimanale, 29 Ottobre 2021. http://www.quotidianosanita.it/allegati/allegato6443633.pdf (2021).
